# Core Level Spectra of Organic Molecules Adsorbed on Graphene

**DOI:** 10.3390/ma11040518

**Published:** 2018-03-29

**Authors:** Abhilash Ravikumar, Gian Paolo Brivio, Guido Fratesi

**Affiliations:** 1 Dipartimento di Scienza dei Materiali, Università di Milano-Bicocca, via Cozzi, 55, 20125 Milano, Italy; gianpaolo.brivio@unimib.it; 2 Dipartimento di Fisica, Università degli Studi di Milano, via Celoria, 16, 20133 Milano, Italy; guido.fratesi@unimi.it

**Keywords:** core excited spectra, organic molecules on graphene, magnetism in graphene

## Abstract

We perform first principle calculations based on density functional theory to investigate the effect of the adsorption of core-excited organic molecules on graphene. We simulate Near Edge X-ray absorption Fine Structure (NEXAFS) and X-ray Photoemission Spectroscopy (XPS) at the N and C edges for two moieties: pyridine and the pyridine radical on graphene, which exemplify two different adsorption characters. The modifications of molecular and graphene energy levels due to their interplay with the core-level excitation are discussed. We find that upon physisorption of pyridine, the binding energies of graphene close to the adsorption site reduce mildly, and the NEXAFS spectra of the molecule and graphene resemble those of gas phase pyridine and pristine graphene, respectively. However, the chemisorption of the pyridine radical is found to significantly alter these core excited spectra. The C 1s binding energy of the C atom of graphene participating in chemisorption increases by ∼1 eV, and the C atoms of graphene alternate to the adsorption site show a reduction in the binding energy. Analogously, these C atoms also show strong modifications in the NEXAFS spectra. The NEXAFS spectrum of the chemisorbed molecule is also modified as a result of hybridization with and screening by graphene. We eventually explore the electronic properties and magnetism of the system as a core-level excitation is adiabatically switched on.

## 1. Introduction

Advances in 2D physics were stimulated with the discovery of graphene in 2004 [1]. Graphene is a two-dimensional allotrope of sp${}^{2}$ hybridized carbon with a honeycomb lattice structure presenting several interesting properties and has been studied extensively in the recent past [2,3,4,5,6]. Graphene shows a linear dispersion of band structure with the valence and conduction bands intersecting at the *K* and $K^{\prime }$ points of the first Brillouin zone corresponding to the Fermi level [3]. This results in several interesting properties such as ballistic transport [1,7,8], the anomalous integral and the half integral quantum Hall effect [9,10] and high charge mobility due to relativistic Dirac fermions [10]. With such high electron mobilities (∼20,000 cm${}^{2}$/V·s) and high switching speeds, graphene-based field effect transistors seem to have the potential to surpass current silicon-based electronics [11,12,13]. However, the absence of a bandgap in graphene results in a low on/off ratio and reduces the output power gain [14,15]. Several doping methodologies have been proposed to overcome this limitation of graphene such as: electrostatic doping due to interaction with a substrate [16,17], hetero-atom substitutional doping by B or N atoms [18,19,20,21] or by covalent functionalization of organic molecules on graphene [22,23,24,25,26]. The effect of molecular adsorption is found to play a key role in determining the electronic and magnetic properties of graphene systems. Furthermore, these graphene/molecule interfaces also have significant implications in the field of organic photovoltaics, molecular electronics, surface catalytic dynamics, gas sensors and femtochemistry [27,28,29,30,31], where the interfacial charge transfer dynamics become relevant. To be exploited fully, this calls for a thorough characterization of the organic/graphene interface at the sub-nm length scale, which requires the capability to link experimental observations to material properties at the atomistic level.

Surface science techniques are very apt to the study of these hybrid interfaces, including experimental methods, which involve core excitation of the organic molecule. Among these, X-ray Photoemission Spectroscopy (XPS) and Near Edge X-ray Absorption Fine Structure (NEXAFS) are element sensitive and give information regarding the chemical bonding environment, preferential adsorption orientations of the molecular layer exploiting the dependence of spectra on the direction of the incoming photon field in relation with the transition dipole moments [8,32,33,34,35]. Charge transfer rates can be studied by the core hole-clock method where the core hole lifetime is used as a reference to determine ultrafast charge transfer times [36,37] with femtosecond resolution. In such core-level techniques, understanding the nature of the molecular interaction with the substrate upon core excitation plays a crucial role.

Density Functional Theory (DFT) [38,39,40] has become the preferred framework to theoretically treat core-excited hybrid systems due to efficient use of atomic potentials, which model the localized core hole by a static positive charge. One can simulate the final state of a X-ray photoemission experiment by a pseudopotential generated without a core electron (Full Core Hole (FCH)) [41]. The theoretical model used to simulate the NEXAFS spectra is the transition potential approach [42,43], where one considers a partial occupation at the core hole (Half Core Hole (HCH)). The occupation of the final state is typically neglected in order to avoid computing every possible transition individually, which may become a formidable task especially for photon energies above the ionization threshold. This method has been successfully validated for a variety of systems [35,44,45,46,47,48].

Most of the previous studies relating to organic molecules/graphene interfaces focus on the electronic and magnetic properties of the system in the ground state or with valence excitations [25,49]. However, experimental works characterizing core-level excited interfaces are raising growing interest; for example, physisorbed systems such as bipyridine/graphene/Ni(111) [37] and terephthalic acid/graphene/Ni(111) [50] interfaces have been studied for their relevance in organic thin-film research. Inorganic covalent defects in the form of hydrogenation and bromination have also been explored by XPS and NEXAFS to provide relevant insight into the effect of covalent doping of graphene [32]. The N 1s NEXAFS spectra of dipolar molecules on graphene have been recently measured and computed [51,52] and the effect of a support considered for the C 1s spectra of graphene [53]. These and similar works stimulate understanding the modifications of the electronic structures of such interfaces upon excitation and its influence on the spectra. In this work, we aim to provide a theoretical spectroscopic understanding of how the nature of the interaction of core-excited organic adsorbates on the graphene interface influences the NEXAFS and XPS spectra. We take two simple cases with a small molecule (pyridine, C${}_{5}$H${}_{5}$N) physisorbed on graphene and its corresponding radical covalently adsorbed, as two paradigmatic examples. The role of graphene in the adsorption process in terms of the X-ray photoemission and absorption spectra is studied along with a discussion on the electronic and magnetic properties of these systems upon creation of a core hole.

## 2. Materials and Methods

We perform first principle calculations based on Density Functional Theory (DFT) [38,39,40] within the Generalized Gradient Approximation (GGA) setup using the Perdew–Burke–Ernzerhof (PBE) exchange correlational functional [54,55] and a plane wave basis set as implemented in the Quantum Espresso simulation suite [56,57]. We use ultra-soft pseudopotentials [58] within the RRKJ recipe [59] for ground state calculations. The effect of the non-local van der Waals interaction, which plays an important role in our system, is compensated using Grimme correction [60]. We construct a 5×7 graphene supercell with a low adsorbate concentration of $6.69\times 10^{-3}$ Å${}^{-2}$ to ensure minimum inter-molecular interactions. The separation of the periodic images in the *z* direction orthogonal to the surface plane is 15 Å. The plane wave kinetic energy cutoff is converged to 42 Ry, and those on the energy and force are $10^{-4}$ a.u. and $10^{-3}$ a.u., respectively. A 3×2 Γ-centered surface k-point mesh is used for system relaxation and total energy calculations. For the density of states calculations, a denser 9×6 Γ-centered k-point mesh is used. The simulation parameters are verified for a free-standing graphene case, and the lattice constant calculated was 2.462 Å, which is in good agreement with the experimental value of 2.46 Å [61].

For calculating the NEXAFS spectrum, one needs to explore the transitions of the core excited electron to unoccupied bound states. This is simulated by considering a fractional core hole (Half Core Hole (HCH)) pseudopotential, which is better suited to describe the synergy of the core-excited electron with the hole left behind. This model has been introduced in the transition potential approach [42,43] and has been successfully employed to various adsorbed systems and interfaces [44,48,62,63]. The calculation of the matrix elements describing the transitions is done using the XSpectra tool [64] within the Quantum Espresso platform, which uses the Lanczos algorithm [65] to efficiently compute the final states. To calculate the XPS spectra, we use the Full Core Hole (FCH) pseudopotential and make use of the ΔSCF (Self-Consistent Field) method [66]. We consider a charged cell for our calculations, which implies a final state of a photoemission experiment where the core electron is excited to a vacuum state, leaving a positively-charged system behind. This describes the system before the possible transfer of an additional electron from the environment [26,37].

## 3. Results and Discussion

### 3.1. Adsorption Configurations

**Figure 1:**
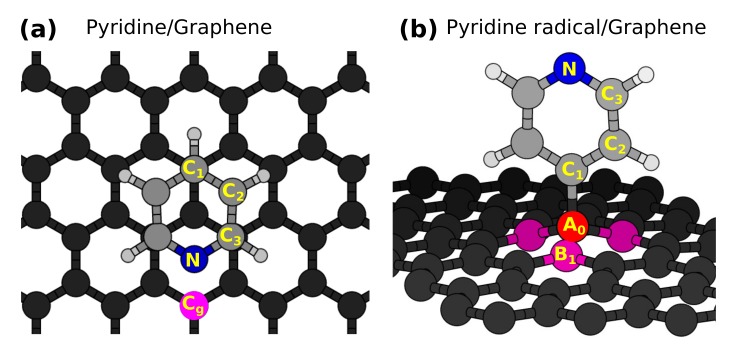
The stable configurations of (**a**) pyridine on graphene and (**b**) the pyridine radical on graphene. C1, C2 C3 are the three inequivalent C atoms of the molecule in the gas phase; the blue sphere stands for the N atom and the smaller gray ones for the H atoms. C${}_{g}$ in (**a**) (magenta) is one of the carbon atoms of graphene close to the N atom of the molecule for which Near Edge X-ray absorption Fine Structure (NEXAFS) spectra are calculated. In (**b**), A${}_{0}$ is the C atom of graphene that participates in the covalent bond with the molecule. By B${}_{1}$, we label the C atoms that are the nearest neighbors to the A${}_{0}$ site and that belong to the other inequivalent sublattice of graphene.

We start by describing the adsorption configurations of the two selected organic moieties on graphene: Pyridine (C${}_{5}$H${}_{5}$N) interacts mainly via weak van der Waals forces with graphene and the pyridine radical chemisorbs on graphene. The most stable configuration of the pyridine/graphene case occurs when the pyridine ring is parallel to the graphene surface and is located above a C atom of graphene (similar to an AB configuration of graphite). The N atom of the molecule is at the center of a graphene ring as shown in Figure 1a. The three inequivalent carbon atoms of the molecule are labeled as C1, C2 and C3. We also choose a C atom of graphene close to the adsorption site (labeled C${}_{g}$) whose purpose will be discussed later. The pyridine radical (C${}_{5}$H${}_{4}$N) chemisorbs on graphene and is oriented perpendicular to the graphene surface as shown in Figure 1b. The covalent adsorption of the molecule with the C atom of graphene (labeled A${}_{0}$) distorts the graphene lattice structure, raising the A${}_{0}$ site atom along with its nearest neighbors (labeled B${}_{1}$ and shown in magenta) with respect to the graphene plane, as shown in Figure 1b. These are general results for covalently-bonded species, from the simple H atom [67] to other chemisorbed organic molecules on graphene [25,49]. Details about adsorption energies, bond lengths and intermediate adsorption configurations have been discussed in our previous work [26].

### 3.2. X-Ray Photoemission

X-ray photoemission experiments allow one to measure the Binding Energy (BE) of the core electrons, in our case most importantly, that of the C 1s. Values of BE are sensitive to the local bonding environment and therefore rich in chemical information. Theoretically, the BE is defined as the energy difference between the system in the ground state and the one with the core electron removed, i.e., in the presence of a core hole. Absolute BEs are difficult to address numerically, but the most important information is often contained in the relative BEs of atoms of the same species, that is the Core Level Shifts (CLS) with respect to a reference value. Their determination can proceed straightforwardly by electronic structure calculations based on all-electron models, but also on more widespread pseudopotential codes. Indeed, it is sufficient to evaluate the difference in energy of the system, when a modified pseudopotential including a 1s core hole (FCH) replaces the standard one at particular atomic sites [41]. In this work, we take the average BE of the C atoms of graphene as a reference so that the CLS${}_{i}$ of the *i*-th C atom is obtained as:

(1) $${\mathrm{CLS}}_{i}=E_{i}^{\mathrm{FCH}}-{\langle E^{\mathrm{FCH}}\rangle }_{gr},$$

where $E_{i}^{\mathrm{FCH}}$ is the total energy of the system with the core hole at the *i*-th atom and ${\langle E^{\mathrm{FCH}}\rangle }_{gr}$ the average of such values across the atoms of graphene in the supercell (70 in our case). To better compare with the free molecule in the forthcoming discussion, we alternatively take as a reference the average across the C atoms of pyridine, ${\langle E^{\mathrm{FCH}}\rangle }_{mol}$.

**Figure 2:**
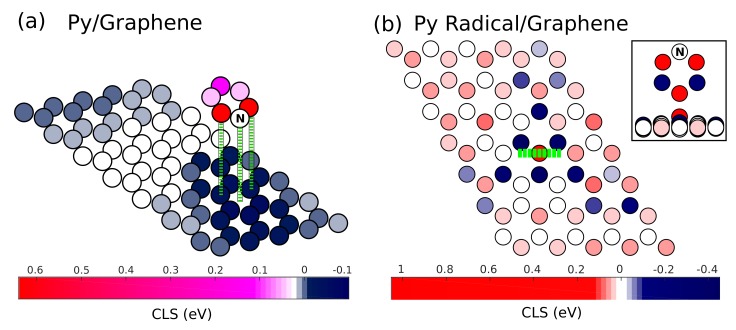
The Core Level Shifts (CLS) with respect to the C 1s edge of graphene is plotted for the two cases: (**a**) Pyridine/graphene and (**b**) the pyridine radical/graphene. The inset of (**b**) shows a side view centered on the molecule.

We can now discuss the effect of core exciting an electron from the C 1s edge of the system and understand the effect of molecular adsorption on the binding energies of the system in terms of the core level shifts. Figure 2a shows as a color map the C 1s CLSs of the pyridine/graphene case, calculated with respect to the average C 1s energy of graphene atoms. Values for selected atoms are tabulated in Table 1, whereas all results can be observed in the Supplementary Materials. The N atom of the molecule is not considered here as the N 1s ionization potential falls in a different excitation energy range. By observing the CLS from Figure 2a, we see that the binding energies of the molecule are larger than the substrate ones by ∼0.3 eV on an average. This observation is consistent with the more efficient screening occurring within graphene than at the molecule, where the removal of the core electron is more expensive. The effect of physisorption of pyridine on graphene reduces the binding energy of the C atoms of graphene close to the adsorption site as the molecular electrons also participate in the screening. For pyridine, this appears to be especially effective around the N atom, as can be observed by looking at the CLSs of the graphene atoms close to it. The smallest CLS indeed is found for the atom of graphene that we labeled $C_{g}$ (shown in Figure 1a), which amounts to −0.06 eV. As for the pyridine molecule, it is instructive to compare the CLSs with respect to the results of the free species. For such a comparison, we consider in both cases the CLSs taken with respect to the molecule (see the values in parentheses in Table 1. Noticeably, these are practically equal to those of the free molecule, testifying that the influence of the screening by graphene substrate is equally effective for a core hole at any molecule C atom that is about the same distance from the substrate. We can therefore conclude that physisorption does not significantly alter the BE of the system and only influences the substrate atoms close to the adsorption site.

The CLSs for the chemisorbed case of pyridine radical/graphene are shown in Figure 2b. We recall that when graphene participates in chemisorption by forming a covalent bond at the A${}_{0}$ site, the corresponding $p_{z}$ orbital is removed from the π system in forming bonding/antibonding orbitals with the adsorbate [25]. Consequently, this site is the one that is expected to differ significantly in its electronic properties from unperturbed graphene. Indeed, the A${}_{0}$ site C atom of graphene has a very large and positive CLS, with the C 1s binding energy larger by ∼1 eV than average, testifying a lower capability of the system to screen a perturbation at this site. Conversely, the CLS on the B${}_{1}$ site C atoms (see Figure 1b) close to the adsorption site is significantly negative, although lower in magnitude (∼−0.4 eV). As one moves away from the adsorption site, one sees that the sign of the CLS of atoms belonging to the B sublattice keeps the negative sign found for the closest ones (B${}_{1}$) with magnitude decreasing with distance, whereas the A sublattice atoms show non-negative CLS without following a clear trend. We remark that atoms that appear farthest from the adsorption sites are however affected by the presence of periodically-repeated units as of the supercell approach. We refer to the Supporting Materials for additional information regarding the CLS values.

For the pyridine radical, the CLSs are on average positive, indicating a larger BE than the graphene atoms similar to the case of the physisorbed molecule. However, the difference with the graphene average is now smaller, which implies the core hole at the molecule being more effectively screened by graphene. In this respect, it is helpful to compare the CLSs taken with respect to the molecular average, as we move from the gas phase pyridine molecule to the chemisorbed radical (see Table 1). In particular, the difference in the CLS of C2 and C3 upon physisorption is practically unaffected (as discussed earlier), but as the molecule is chemisorbed, such a difference increases from 0.59 eV to 0.69 eV. This can be understood since the height over graphene is approximatively equal for C2 and C3 atoms in the physisorbed case, but it is larger by 1 Å for the C3 atom of the chemisorbed radical, resulting in a less efficient screening and a larger BE. Remarkably, the C1 atom that is closest to graphene also shows an increase of BE upon chemisorption, consistent with the low capabilities of the A${}_{0}$ site to screen the core excitation (recall the large CLS of the A${}_{0}$ atom).

### 3.3. X-Ray Absorption

We now discuss the Near Edge X-ray Absorption Fine Structure (NEXAFS) spectra calculated for gas phase pyridine and further inspect the NEXAFS spectra for the organic/graphene systems to understand the effect of adsorption on the core excited spectra. The N 1s NEXAFS spectra have been shown in the Supplementary Materials, which do not differ significantly from the C 1s shown here and therefore are not discussed in this section.

**Figure 3:**
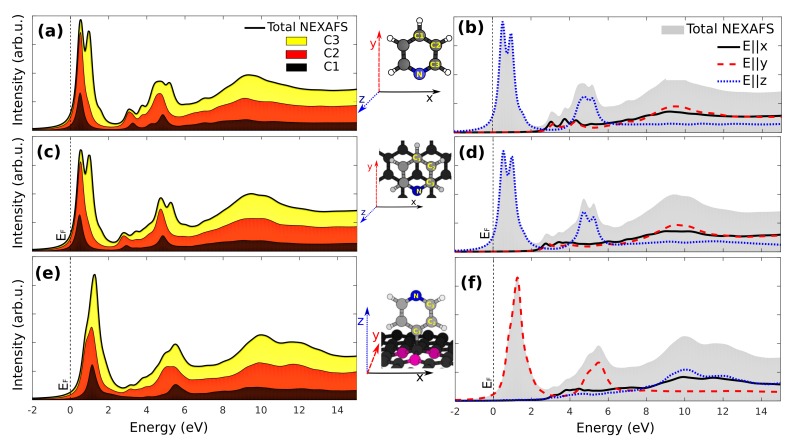
NEXAFS spectra at the C 1s edge for (**a**) the gas phase pyridine molecule showing the contribution of the inequivalent C atoms towards the final spectra and (**b**) the spectra for different electric polarizations are shown; (**c**,**d**) show corresponding data for the pyridine/graphene interface and (**e**–**f**) for the pyridine radical on graphene. The photon energy scale is aligned to the energy required to promote a C 1s electron to the graphene Fermi level. Panels (**a**,**b**), missing this reference, are aligned to match the pyridine/graphene case.

Let us first discuss the C 1s NEXAFS of gas phase pyridine, which is shown in Figure 3a,b. As for the analysis of the CLSs, the absolute energy scale is not accessible by our method [35], so the origin of the energy axis is fixed arbitrarily in the figure to align to the molecule/graphene case discussed later. Figure 3a shows the total, spherically-averaged C 1s NEXAFS of gas phase pyridine decomposed into the contributions from the inequivalent C atoms (C1, C2 and C3 as shown in the inset). A first feature consisting of a double peak split by 0.44 eV can be observed. This originates from the 1s → LUMO transitions that are at different energies following the different BE of the corresponding atoms that we reported in Table 1: in particular, at high energy, one finds the contribution from the C3 atom, which displays the highest BE. Polarized NEXAFS spectra, as accessible at synchrotron radiation facilities, further provide information on the molecular orbital symmetry and molecular orientation. In this respect, Figure 3b shows the same C 1s NEXAFS spectra of gas phase pyridine, now resolving the different polarizations of the incoming photon field. The molecular LUMO is a π state, and as such, the first feature only appears for the photon electric field perpendicular to the pyridine ring (*z* axis). Resonances resulting from transitions to states with the σ character lying in the continuum of excitations can be seen at ∼10 eV higher energy (*x* and *y* photon field directions). These observations are in excellent agreement with previous calculations and experiments [68].

We now discuss the effect of physisorption of the pyridine/graphene system on the C 1s NEXAFS spectra shown in Figure 3c,d, presenting the atom-wise contributions and polarized spectra, respectively. Here, the energy for a C 1s $\to E_{F}$ transition, $E_{F}$ being the Fermi energy of the system, can be taken as a reference. The nature of the contributions is very similar to the free molecule case, indicating that the weak van der Waals coupling of the molecule with graphene does not significantly affect the NEXAFS spectra. The same applies to Figure 3d showing the C 1s NEXAFS spectra for different photon polarizabilities. Here, the in-plane molecular axis spans the surface *x* and *y* directions, so that excitation to the molecular LUMO occurs in an experiment with the photon field in the *z* direction orthogonal to the surface (commonly referred to as *p*-polarization [69]).

The NEXAFS spectra for the covalently-adsorbed pyridine radical show instead interesting changes with respect to the free molecule, as we report in Figure 3e,f. The increased splitting of the CLS of C2 and C3 atoms upon chemisorption, remarkably, does not translate to an enhanced splitting of the two peaks within the LUMO feature in the NEXAFS spectrum. Conversely, that merges into a single main peak whose transition dipole moment is perpendicular to the pyridine plane showing a peak on the low-energy side, mostly as a result of the increased CLS of the C1 atom (seen in Table 1).

**Figure 4:**
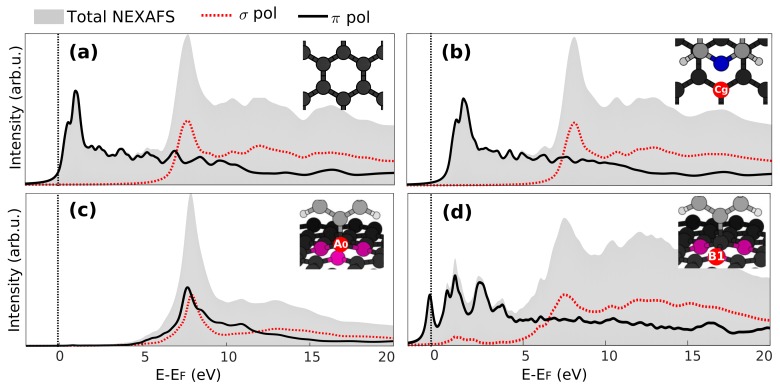
Total NEXAFS spectra at the C 1s edge and the components resolved depending on the photon electric field direction (*p*-polarization, transition to π states, *s*-polarization, transition to σ states) for (**a**) pristine graphene; (**b**) a C atom of graphene (C${}_{g}$) close to the physisorbed pyridine (**c**). The bonding C atom of graphene participating in the covalent bond with the pyridine radical (A${}_{0}$ site), and (**d**) a nearest neighbor C atom of the bonding site (B${}_{1}$ site).

We now look at the NEXAFS spectra of graphene and the influence of molecular adsorption on the latter. Figure 4a shows the NEXAFS spectra calculated for pristine graphene. Two main structures are observed, in good agreement with previously-reported studies [32]. The first feature at lower energy corresponds to excitation to the π electron system close to the Fermi level that occurs with the photon electric field along the graphene normal *z* (*p*-polarization). The second one, at about 7.7 eV, is instead due to a transition to σ states that can be probed by the photon electric field in the graphene plane (*s*-polarization) that we compute as the average of the *x* and *y* surface azimuths. One can observe by comparing these results to those in Figure 4b that physisorption of pyridine does not significantly alter the NEXAFS spectra of graphene. There, we have chosen to report the NEXAFS spectrum calculated for the C${}_{g}$ atom of graphene since this is the one that experiences the largest CLS change as compared to the other graphene atoms (as shown in Figure 2a).

The effect of chemisorption of pyridine radical presents a significant effect on the NEXAFS of graphene. The A${}_{0}$ site C atom of graphene participates in the covalent bond with the pyridine radical by donating its $p_{z}$ orbital and forming bonding states at energies at least 4 eV below $E_{F}$ [25]. The corresponding antibonding orbital can be found in the unoccupied states probed by NEXAFS at 7.7 eV, as can be seen by the peak for the *p*-polarized spectrum in Figure 4c. Conversely, the intensity close to the Fermi level completely vanishes due to the chemisorption. It is also interesting to notice that the spectra in *p*- and *s*-polarization are very similar, consistent with the sp${}^{3}$ hybridization at this sites. An intermediate situation occurs for the NEXAFS spectra at the B${}_{1}$ site C atoms: these retain, to some degree, the resemblance with free standing graphene, as seen in Figure 4d, especially as the spectra in *s*-polarization are compared. The empty states corresponding to the $p_{z}$ orbitals (*p*-polarization) are perturbed by the orbital missing at the A${}_{0}$ site, but still contribute to the spectral intensity at low energies. Finally, the presence of some states close to the Fermi level (∼1 eV) also in the *s*-polarized spectra is a result of the distortion of the graphene lattice and hybridization of σ and π orbitals.

### 3.4. Core-Excited Electronic and Magnetic Properties

**Figure 5:**
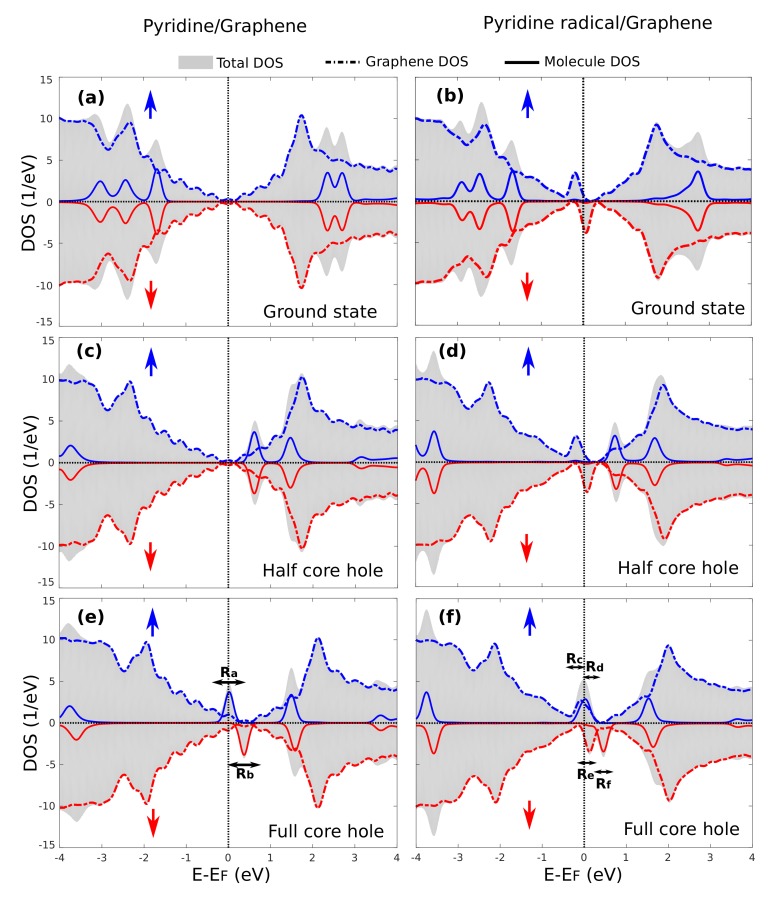
Electronic Densities Of States (DOS), total and projected onto the orbitals of the molecule and on graphene, for ground state (**a**) pyridine/graphene and (**b**) pyridine radical/graphene, half core hole (**c**) pyridine/graphene and (**d**) pyridine radical/graphene and full core hole (**e**) pyridine/graphene and (**f**) pyridine radical/graphene. Positive/negative values indicate spin-majority/-minority populations, respectively. Arrows in (**e**,**f**) mark energy ranges for reference in Figure 6.

We now discuss how the core-level excitation influences the electronic and magnetic properties of the two systems. For the following qualitative analysis, we concentrate on one specific case out of the possible 1s excitation sites; given the large number of inequivalent C atoms that could be considered, we select here the N atom in the molecule. It is instructive to switch on the perturbation adiabatically by performing calculations including a N 1s hole of increasing magnitude λ, also considering that λ=0.5 corresponds to the HCH method employed for NEXAFS simulations and λ=1.0 to the FCH one of use for XPS analysis. The corresponding electronic Densities Of States (DOS) are shown in Figure 5. There, spin-majority and spin-minority components are plotted separately (the latter with an inverted sign), and the projected DOS on the adsorbate and on graphene are shown. We briefly recall first the properties of the systems in the ground state [26]. The physisorbed system is non-magnetic due to a weak van der Waals interaction of the molecule with graphene, as shown in Figure 5a; whereas chemisorption of a molecule distorts the lattice symmetry of the substrate and graphene donates one of its $p_{z}$ electron to participate in the covalent bond [25]. This unbalance between the graphene sublattices results in a magnetic solution with a 1 $\mu_{B}$ magnetic moment carried by mid-gap states of graphene that are visible in the DOS close to the Fermi level, which have been well studied in previous works [25,26,49]. The molecular orbitals are quite far away from the Fermi level in both cases, with the LUMO appearing in the DOS projected on the molecule at an energy larger than $E_{F}+2$ eV, as seen in Figure 5b.

**Figure 6:**
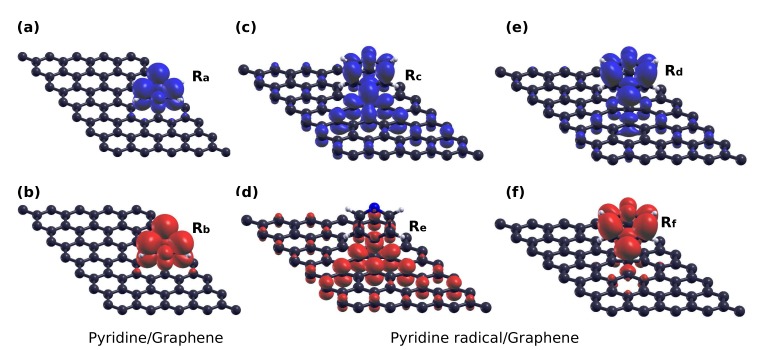
Local density of states integrated in energy (Integrated Local Densities Of States (ILDOS)) over the energy intervals marked by arrows in Figure 5e,f. (**a**,**b**) for pyridine/graphene, around the molecular LUMO for the (**a**) spin-majority and (**b**) spin-minority channels; (**c**,**d**) the mid-gap/LUMO hybrid states in the spin-majority channel for pyridine radical/graphene and (**e**,**f**) the spin-minority mid-gap and LUMO states.

Figure 5c,e shows the DOS of pyridine/graphene for the half core hole and full core hole final states, respectively. As a response to the creation of the core hole, the molecular orbitals are shifted to lower energies due to Coulombic interaction to screen the positive core. In Figure 5c, where a 0.5 core charge corresponds to the transition-potential of X-ray absorption simulations, we can see that the LUMO comes closer to $E_{F}$, but is still unoccupied (at energy ∼$E_{F}+0.7$ eV). The distribution of electronic charge between the two subsystems, which we compute by Löwdin population analysis, is unchanged within 0.01 e. The system is still non-magnetic as it was in the ground state. However, when the full core hole is created and the core electron is photoemitted from the system, the molecular LUMO shifts close to the Fermi level. The spin-up LUMO shifts partially below the Fermi level, and the spin-down LUMO remains above, resulting in a spin-polarized solution. We can visualize the wave functions of the system in real space by calculating the energy-Integrated Local Densities Of States (ILDOS), where the integral is performed over the energy interval of interest. This has been done for the ranges highlighted in Figure 5e by the arrows labeled (R${}_{a}$) and (R${}_{b}$) for the two spin components. The results are reported in Figure 6a,b for the spin-majority and spin-minority channel, confirming these peaks are dominated by the LUMO with a minor contribution by graphene (slightly larger in the spin-majority case). Compared to the ground state, we find an excess of 0.33 e on the molecule, withdrawn from graphene, which enables the LUMO to participate in screening the perturbation. As a consequence, the Dirac cone shifts to higher energies as appreciated in Figure 5e. We remark that such a shift is strongly affected by the finite density of excited molecules and would decrease by increasing the simulation cell. Overall, the system reaches a partially-magnetic solution with a magnetic moment of 0.3 $\mu_{B}$ on the molecule.

For a chemisorbed system, in the HCH case, as shown in Figure 5d, the system is magnetic due to the presence of spin-dependent mid-gap states of graphene similarly to the ground state case. The molecular LUMO shifts to lower energy values (∼$E_{F}+0.9$ eV) and is equal for the two spin populations, but does not take part in the screening process. Nevertheless, since the mid-gap state hybridizes with molecular ones, the molecular Löwdin charges increase by 0.09 e with respect to the ground state even if this does not correspond to a filling of the LUMO. Upon creation of a full core hole, as shown in Figure 5f, the LUMO shifts to the Fermi level. Here, it hybridizes with the mid-gap states of graphene to form a mid-gap-LUMO hybrid state in the spin up channel. This consists of two states, of which we plot the ILDOS in Figure 6c,e, whose energy ranges are labeled as (R${}_{c}$) and (R${}_{d}$) in Figure 5f. The first one is the bonding combination between them, and is filled; the second one is the anti-bonding combination (see a nodal plane between the molecule and graphene) and is empty, so that the system still remains magnetic, but now, the spin polarization is both on graphene (∼60%) and on the molecule (∼40%). In the spin-down channel, the LUMO does not hybridize with the mid-gap state, as can be visualized by the ILDOS shown in Figure 6e,f whose energy ranges are appropriately marked in Figure 5f as (R${}_{e}$) and (R${}_{f}$). Similar to the physisorbed case, the transfer of electrons to the molecule in order to screen the perturbation amounts to 0.44 e, and results in a shift of the Dirac point of graphene towards the unoccupied states. The addition of a further electron to the valence shell, as in X-ray absorption, would instead preferentially fill the minority spin population, and at self-consistency, the electronic structure changes with the extra electron being shared by the mid-gap and the spin-down LUMO forming hybrid states analogously to the spin-majority one [26].

Before concluding this section, we remark that the shift of the Dirac cone as induced by a localized perturbation (treated by the repeated supercell approach) is a signature of electrons being attracted towards the perturbation from the surrounding graphene, hence neutralizing the system. Electron transfer from graphene to core-excited molecules has indeed been observed for bipyridine adsorbed on graphene/Ni or Au substrates [37] where it occurs on a femtosecond timescale. It is therefore also interesting to look at the formation of the core hole for a neutral system, which is detailed in the Supplementary Materials.

## 4. Conclusions

We have studied the effect of adsorption of core excited organic molecules on graphene by analyzing the core level shifts and the NEXAFS spectra. For the physisorbed pyridine/graphene case, the relative binding energies of the molecule are comparable with those of the gas phase. One observes that the binding energies of the graphene C atoms close to the adsorption site decreases by ∼0.3 eV when compared to those of farther C atoms. As for the chemisorbed pyridine radical/graphene, the BE of C atom of the molecule participating in the covalent bond increases slightly when compared to the gas phase molecule. The C atom of graphene participating in the chemisorption (A${}_{0}$) displays a major difference with an increase in its BE when compared to rest of the graphene atoms by ∼1 eV. Its nearest neighbor graphene atoms (B${}_{1}$) show a smaller, but still significant reduction in BE (∼−0.4 eV).

From the NEXAFS of adsorbed pyridine, we can conclude that physisorption via weak van der Waals forces minimally alters the NEXAFS spectra of the molecule and is comparable to gas phase pyridine. The same is true for the NEXAFS spectra of graphene, which resembles the one of pristine graphene. As for the chemisorbed pyridine radical/graphene case, we find a significant distortion in the NEXAFS spectra of the molecule and especially graphene atoms close to the adsorption site. Following the increase in binding energy of the C atom of the molecule participating in the covalent bond, its contributions to the NEXAFS spectra also shift to higher energies. The loss of the $p_{z}$ electron at the A${}_{0}$ graphene site is seen in the NEXAFS spectra where the π contributions close to the Fermi level vanish and only higher energy transitions are found, not resolvable into $\sigma^{*}$ and $\pi^{*}$ orbital characters; whereas the NEXAFS spectra at the B${}_{1}$ sites show the persistence of these π contributions, however broadened significantly due to molecular screening and graphene lattice distortion.

Upon creation of the core hole, the system gradually reaches a solution, with the LUMO shifting towards the Fermi level. For physisorbed pyridine/graphene, a magnetic moment mostly localized on the molecule is computed. Upon core excitation of the chemisorbed pyridine radical/graphene, the LUMO and the mid-gap state hybridize in the spin-up channel, whereas the spin-down LUMO remains unoccupied and decoupled from graphene mid-gap states. This induces a magnetic moment on the system, which is shared between the molecule and graphene.

## Figures and Tables

**Table 1:** The CLS with respect to the C atoms of the molecule is tabulated for gas phase pyridine along with the CLS with respect to graphene (molecule) for the two cases: pyridine/graphene and pyridine radical/graphene. The CLSs for a few selected atoms of graphene as specified in Figure 1 are also included.

Atom Site	Gas Phase	Pyridine/Graphene	Pyridine Radical/Graphene
	(CLS${}_{\mathrm{mol}}$) (eV)	CLS${}_{\mathrm{gr}}$ (CLS${}_{\mathrm{mol}}$) (eV)	CLS${}_{\mathrm{gr}}$ (CLS${}_{\mathrm{mol}}$) (eV)
C1	(−0.02)	0.26 (−0.06)	0.18 (0.02)
C2	(−0.29)	0.04 (−0.28)	−0.19 (−0.35)
C3	(0.30)	0.64 (0.31)	0.50 (0.34)
C${}_{g}$	-	−0.06 (−0.38)	-
A${}_{0}$	-	-	1.03 (0.87)
B${}_{1}$	-	-	−0.42 (−0.59)

## Supplementary Materials

The following are available online at http://www.mdpi.com/1996-1944/11/4/518/s1: Figure S1: NEXAFS spectra at the N 1s edge showing the contribution for different electric polarizations for (a) the gas phase pyridine molecule, (b) pyridine/graphene and (c) pyridine radical/graphene; Figure S2: (a) Perspective view and (b) top view of pyridine/graphene showing the CLS with respect to the C 1s edge in graphene. Tics on the axes mark atomic coordinates in Å; Figure S3: (a) Perspective view and (b) top view of pyridine/graphene showing the CLS with respect to the C 1s edge in the molecule. Tics on the axes mark atomic coordinates in Å; Figure S4: (a) Perspective view and (b) top view of pyridine radical/graphene showing the CLS with respect to the C 1s edge in graphene. Tics on the axes mark atomic coordinates in Å; Figure S5: (a) Perspective view and (b) top view of pyridine radical/graphene showing the CLS with respect to the C 1s edge in the molecule. Tics on the axes mark atomic coordinates in Å; Figure S6: The left column plots the total DOS, projections on the molecule and on graphene for different core hole magnitudes (q) of the physisorbed pyridine/graphene case. The right column is the same for pyridine radical/graphene case; Figure S7: The spin up and spin down charges on the (a) molecule and (c) graphene for the physisorbed pyridine/graphene case. (b–d) are the same for the chemisorbed pyridine radical/graphene case; Figure S8: The total magnetic moment of the system for the N 1s excitation for different partial core charges for pyridine/graphene and pyridine radical/graphene cases is shown.

## Abbreviations

The following abbreviations are used in this manuscript:

FCH	Full Core Hole
HCH	Half Core Hole
CLS	Core Level Shift
BE	Binding Energy
