# Supplementary material for: Core Level Spectra of Organic Molecules Adsorbed on Graphene
Source: Materials (Basel). 2018 Mar 29;11(4):518. doi: 10.3390/ma11040518 (PMC5951364; doi:10.3390/ma11040518)
Supplement: Supplementary file 1 [file materials-11-00518-s001.pdf]

materials

Article

Supplementary Materials for Core level spectra of organic molecules adsorbed on graphene

Abhilash Ravikumar [1,*], Gian Paolo Brivio [1] and Guido Fratesi [2]

1 Dipartimento di Scienza dei Materiali, Università di Milano-Bicocca, Via Cozzi 55 - 20125 Milano, Italia.
2 Dipartimento di Fisica, Università degli Studi di Milano, Via Celoria, 16 - 20133 Milano, Italia.
* Correspondence: a.ravikumar@campus.unimib.it; Tel.: +39-3451475855

Academic Editor: name
Received: date; Accepted: date; Published: date

S1. NEXAFS spectra at the N 1s edge

The NEXAFS spectra computed at the N 1s edge for the gas phase pyridine, pyridine on graphene and pyridine radical on graphene are shown in Figure S1. The information regarding the molecular orientation can be extracted NEXAFS spectra by the direction of polarization of the incoming x-ray and has been discussed in detail in the main for the C 1s edge. Here we would like to point out that core level excitation at the N 1s edge does not significantly alter the NEXAFS spectra of the molecule.

S2. Core level shifts (CLS)

The core level shifts for the two adsorption configurations have discussed in detail in the main paper. Here we expand the figures reported in the main text to show the CLS values corresponding to each atom with respect to graphene and the molecule. Figure S2 and Figure S3 show each one two views of physisorbed pyridine/graphene, including at the appropriate atomic positions the CLS calculated for each atom with respect to the C 1s edge of graphene (Figure S2) and the molecule (Figure S3). The CLS with respect to the C 1s edge of graphene and the molecule for the chemisorbed pyridine radical/graphene case are analogously shown in Figure S4 and Figure S5, respectively.

S3. Partial core-level occupation for a neutral system

In the main text, we discuss a system which is a final state of a photoemission experiment. Upon core-excitation we consider that the core electron is completely removed to a vacuum state and the system is left positively charged. Another possible final state is that created by X-ray absorption at a photon energy corresponding to the 1s→LUMO transition or when a substrate electron neutralizes the molecule after photoemission. In such a case the system is globally neutral. This has been studied in detail in [1] to explore the possible effects of magnetism which is induced to the system depending on the adsorption character. Here we explore this further by considering a gradual increase of the core hole charge q taking as an example the N atom, and track the electronic properties, screening charges, and magnetism as a function of q.

In the physisorbed pyridine/graphene case, as discussed in the main of this paper and studied in [1], in the ground state, the system is non-magnetic and the Dirac cone is still preserved. In the left column of Figure S6, we plot the total DOS, its projections on the molecule and graphene (with a Gaussian smearing of 0.013 eV) for a gradual increase of the core hole magnitude q.

We observe that as we increase q (which implies a gradual removal of the core electron and the creation of a core hole), The molecular orbitals shift to lower energy values in order to screen the positive core. But until $q \sim 0.5$ screening is operated by graphene only and a non-magnetic solution (with a magnetic moment of no more than 0.02 μB) is found. But for higher values of q, the molecular LUMO shifts below the Fermi level and results in spin-polarized solutions. For $q = 0.6$, the system

magnetic moment is still relatively small with 0.16 μB. Whereas for a full core hole case ($q = 1.0$), the system reaches a magnetic solution with a total magnetic moment of 0.8μB.

In the chemisorbed case of pyridine radical on graphene, the system is magnetic in the ground state with spin-dependent midgap states contributed by graphene [2,3]. In the right column of Figure S6, we plot the DOS of the chemisorbed case for different values of q. For values of $q < 0.6$, the LUMO shifts steadily to lower energies but is still above the Fermi level. When $q = 0.8$, the LUMO splits into spin polarized components and the spin up LUMO begins hybridization with the spin up midgap state of graphene. The spin down LUMO is still uncoupled from the midgap state. For a full core case ($q = 1.0$), both the spin components of LUMO hybridize with the midgap states of graphene and the calculation reaches a non-magnetic solution.

The charge on the molecule and graphene along the two spin components for pyridine/graphene and pyridine radical/graphene is plotted in Figure S7. Variations in this quantity allow us to identify charge displacements towards the excitation region that occur in order to screen the core hole. The total magnetic moment for the physisorbed pyridine/graphene and chemisorbed pyridine radical/graphene can be deduced as the difference in the spin-up and spin-down charges and is plotted in Figure S8. In Figure S7 (a) and (c) we see that the system is non magnetic and the charge along the two spin components are similar for $q < 0.5$ and that charge displacements mostly involve graphene. As the core charge increases further, the charges along the spin components on the molecule split whereas the graphene ones remain similar to each other. This is in correlation with the magnetic moment which is induced on the molecule after $q = 0.5$ as seen in the left column of Figure S6. For the chemisorbed case as shown in Figure S7 (b) and (d), initially the spin components on graphene are split due to the midgap states of graphene. The molecular spin components are relatively similar. As q increases we see that the charges along the two spin components converge to the same value. An interesting behavior occurs for $q = 0.8$: there, the spin up LUMO begins hybridizing with the spin-up midgap state, there is a magnetic moment induced on the molecule (as seen in the right column of Figure S6) and this can be visualized here as an enhanced splitting of the spin-up and spin-down charges, whereas the total charge on the molecule which follows a continuous trend as a function of q. But the system reaches a non-magnetic solution as the hybridization of the LUMO and midgap states continues for higher values of core charge.

1. Ravikumar, A.; Baby, A.; Lin, H.; Brivio, G.P.; Fratesi, G. Femtomagnetism in graphene induced by core level excitation of organic adsorbates. *Sci. Rep.* **2016**, *6*, 24603.
2. Santos, E.J.G.; Ayuela, A.; Sánchez-Portal, D. Universal magnetic properties of sp 3 -type defects in covalently functionalized graphene. *New J. Phys.* **2012**, *14*, 043022.
3. Lin, H.; Fratesi, G.; Brivio, G.P. Graphene magnetism induced by covalent adsorption of aromatic radicals. *PCCP* **2014**, *17*, 2210–2215.

© 2018 by the authors. Licensee MDPI, Basel, Switzerland. This article is an open access article distributed under the terms and conditions of the Creative Commons Attribution (CC BY) license (http://creativecommons.org/licenses/by/4.0/).

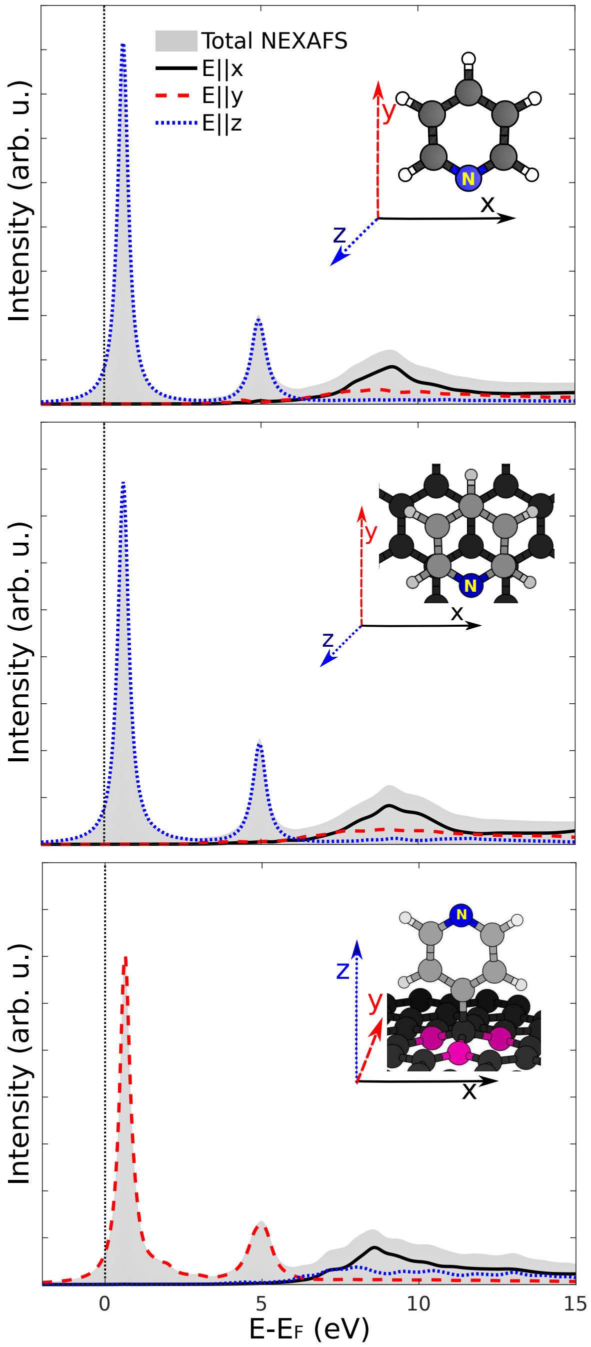

Figure S1. NEXAFS spectra at the N 1s edge showing the contribution for different electric polarizations for **(a)** gas phase pyridine molecule, **(b)** pyridine/graphene, and **(c)** pyridine radical/graphene.

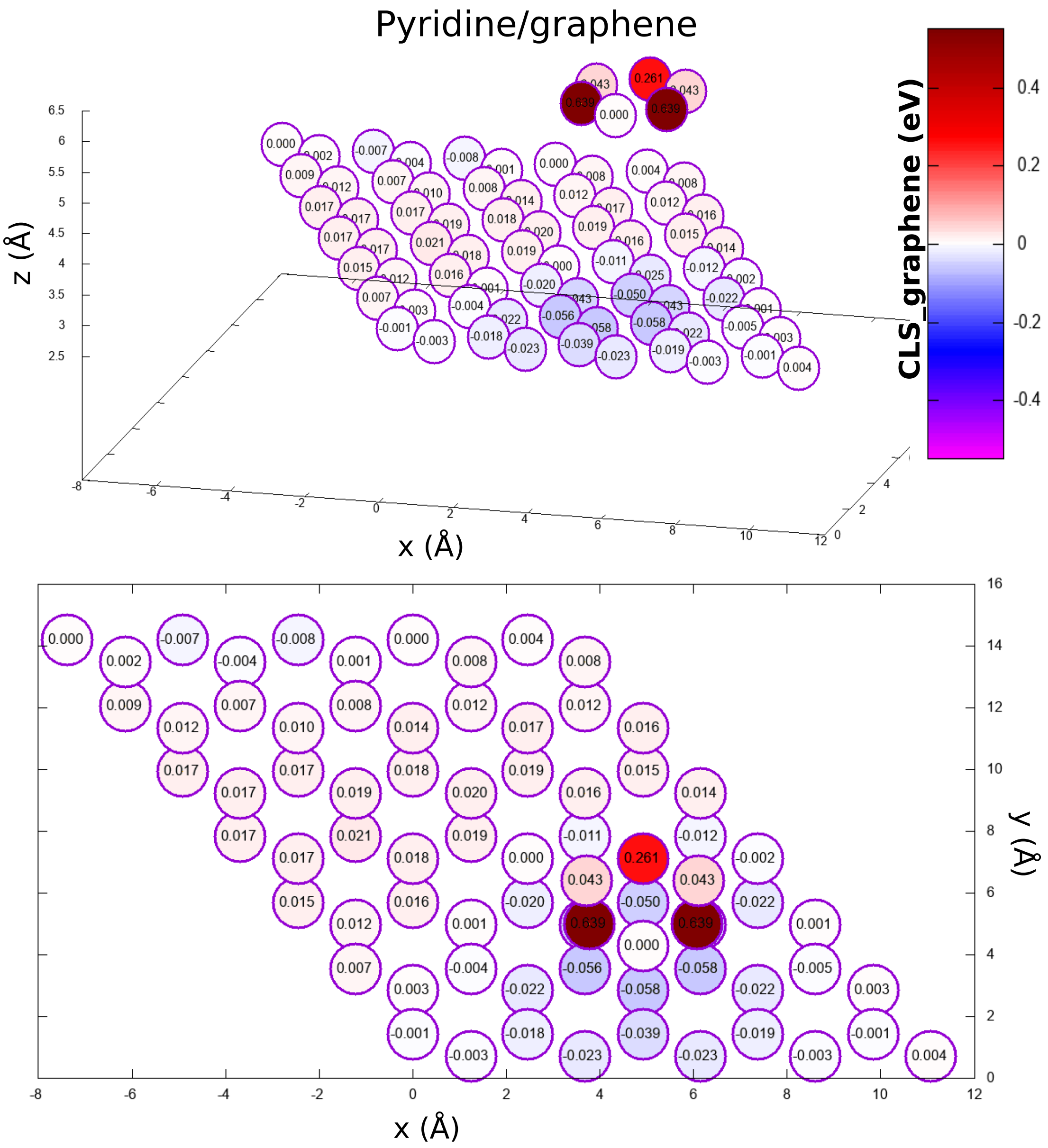

Figure S2. **(a)** Perspective view and **(b)** top view of pyridine/graphene showing the CLS with respect to C 1s edge in graphene. Tics on the axes mark atomic coordinates in Å.

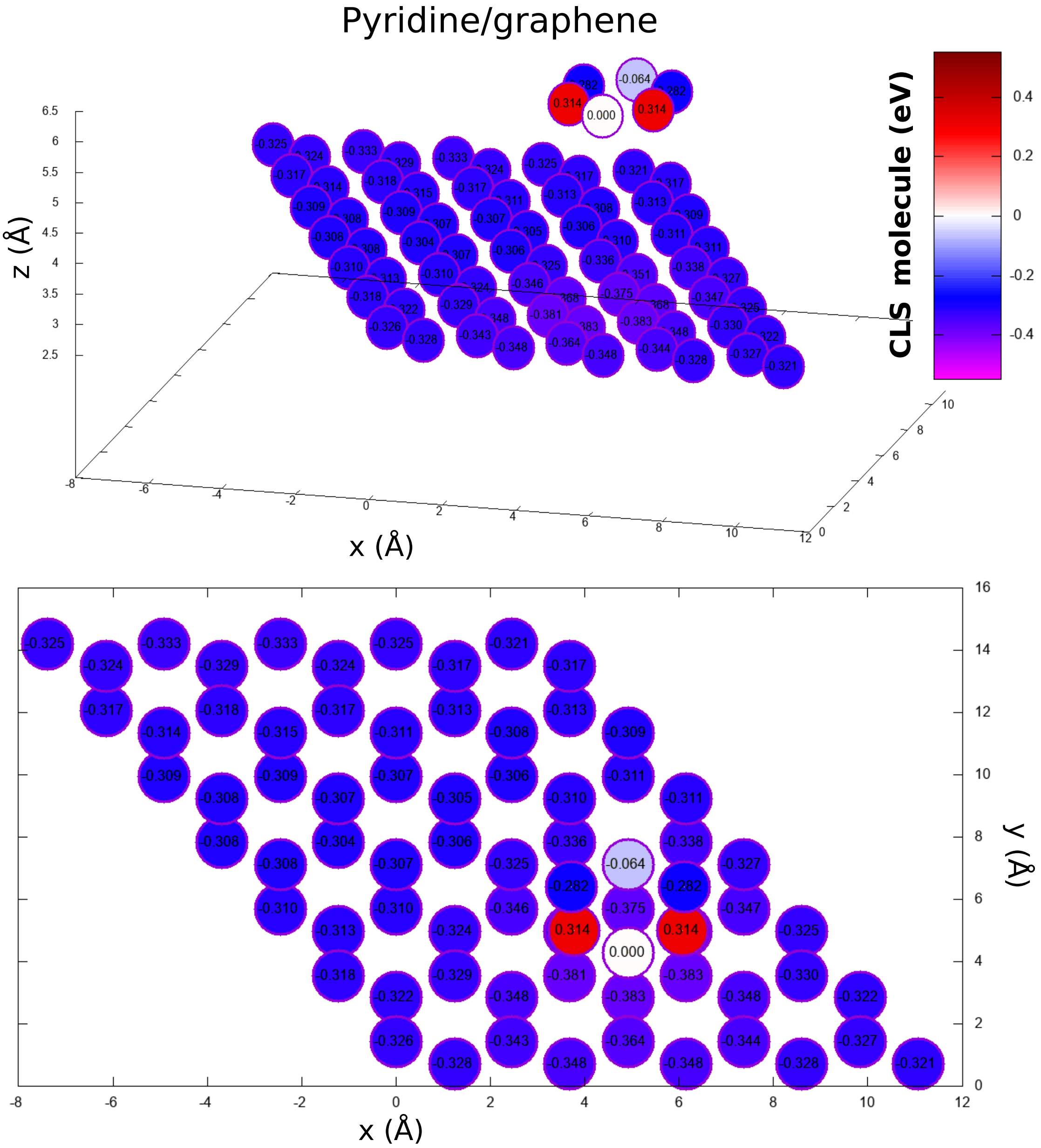

Figure S3. **(a)** Perspective view and **(b)** top view of pyridine/graphene showing the CLS with respect to C 1s edge in the molecule. Tics on the axes mark atomic coordinates in Å.

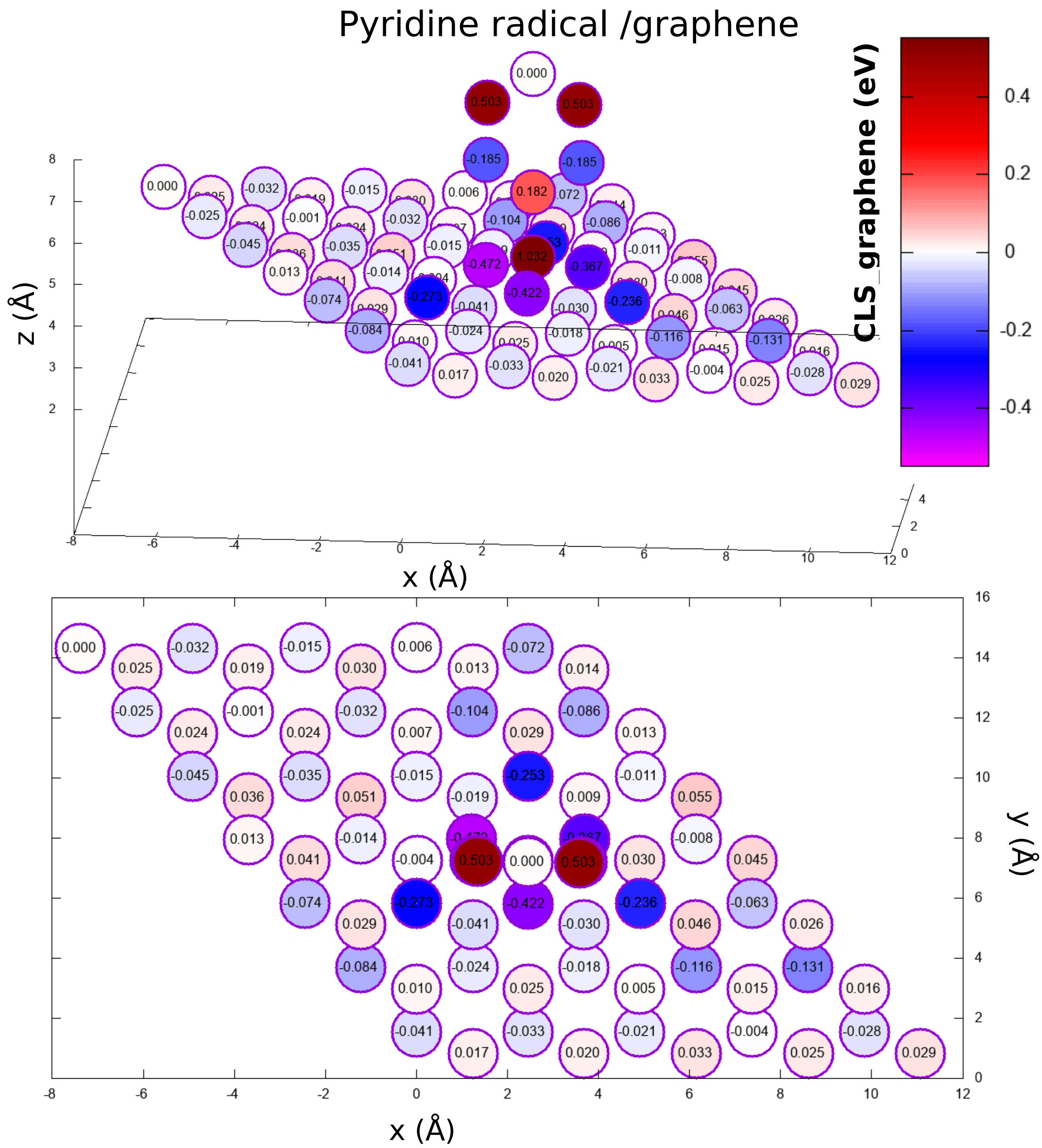

Figure S4. **(a)** Perspective view and **(b)** top view of pyridine radical/graphene showing the CLS with respect to C 1s edge in graphene. Tics on the axes mark atomic coordinates in Å.

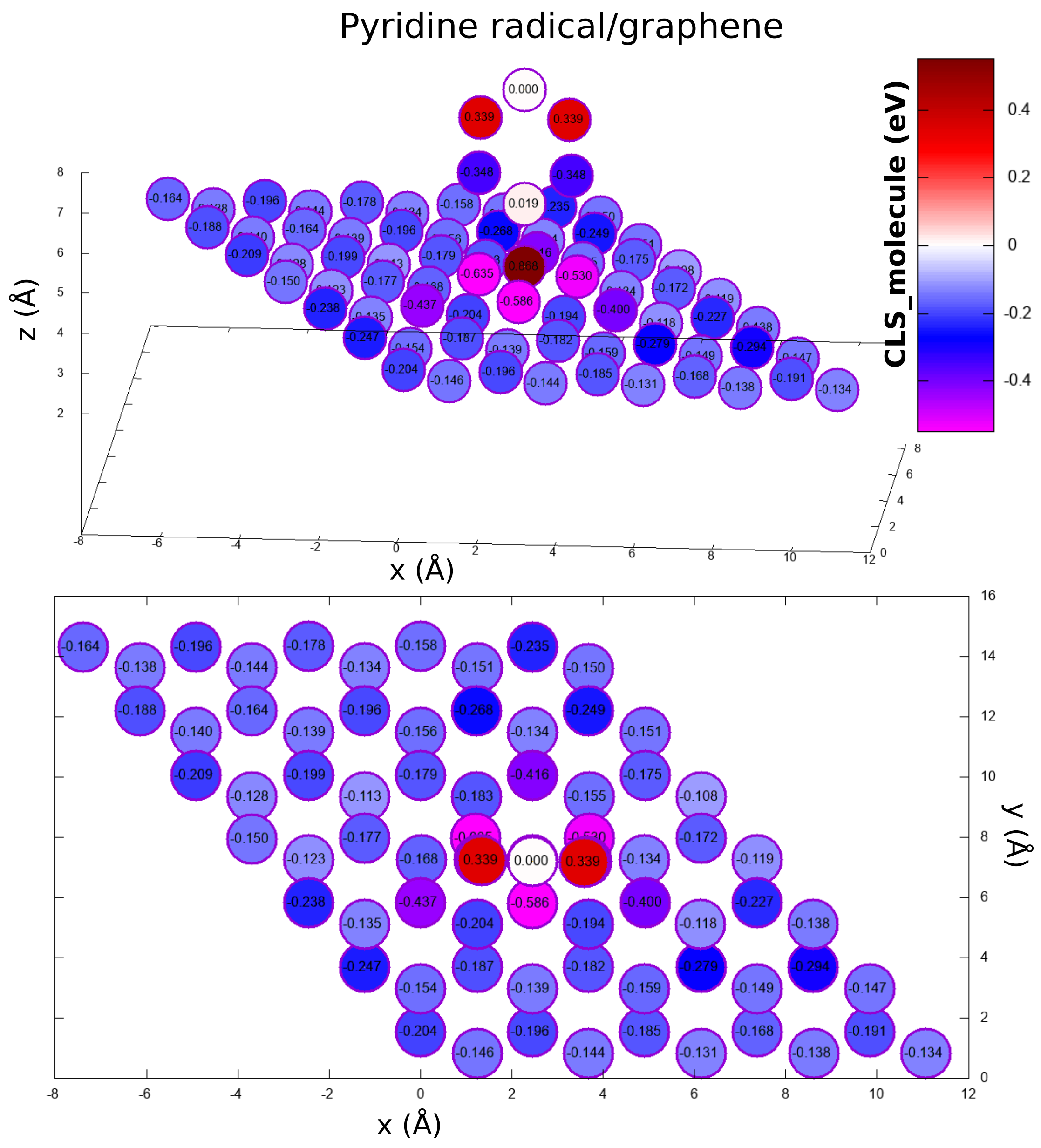

Figure S5. (a) Perspective view and **(b)** top view of pyridine radical/graphene showing the CLS with respect to C 1s edge in the molecule. Tics on the axes mark atomic coordinates in Å.

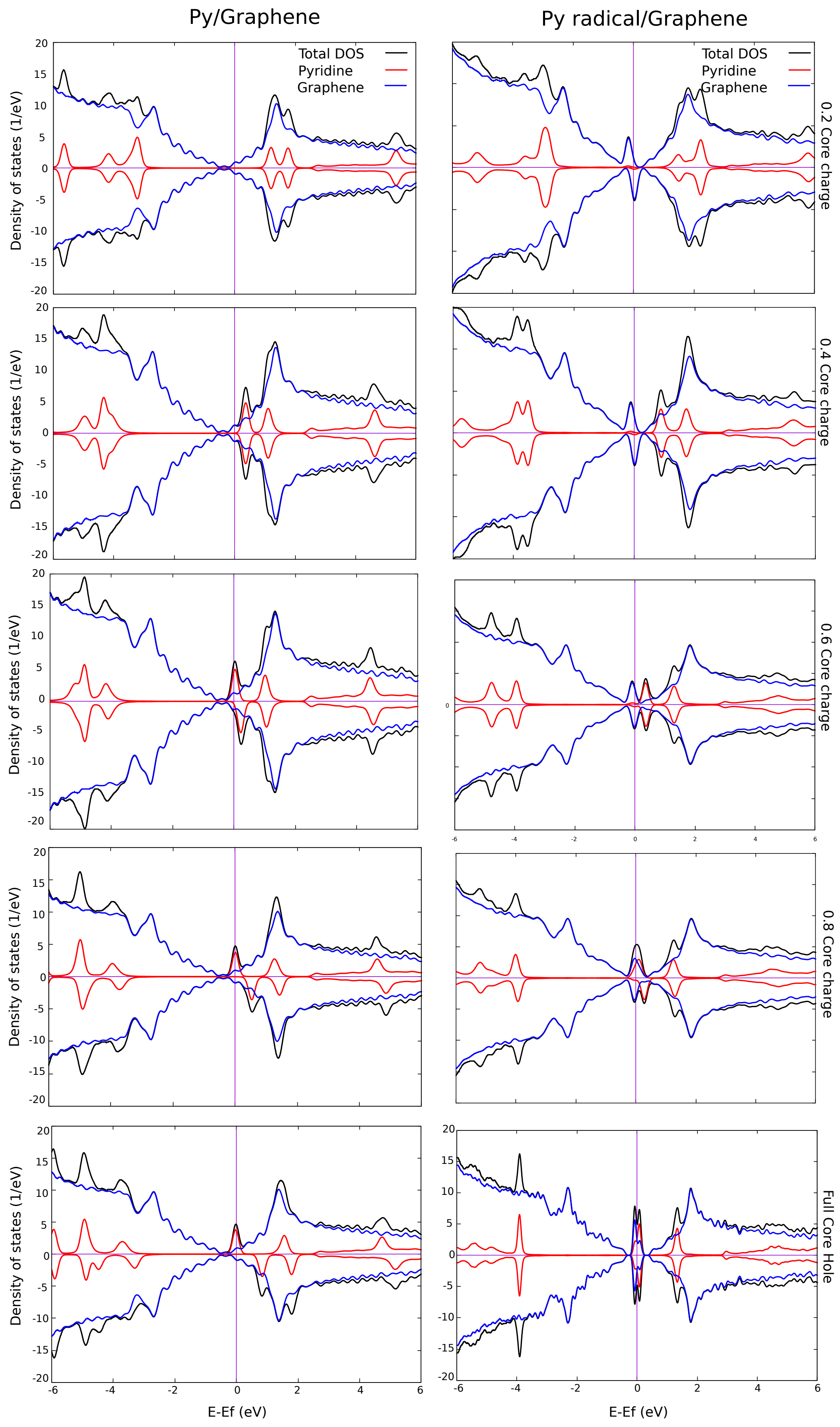

Figure S6. The left column plots the total DOS, projections on the molecule and on graphene for different core hole magnitudes (q) of the physisorbed pyridine/graphene case. The right column is the same for pyridine radical/graphene case.

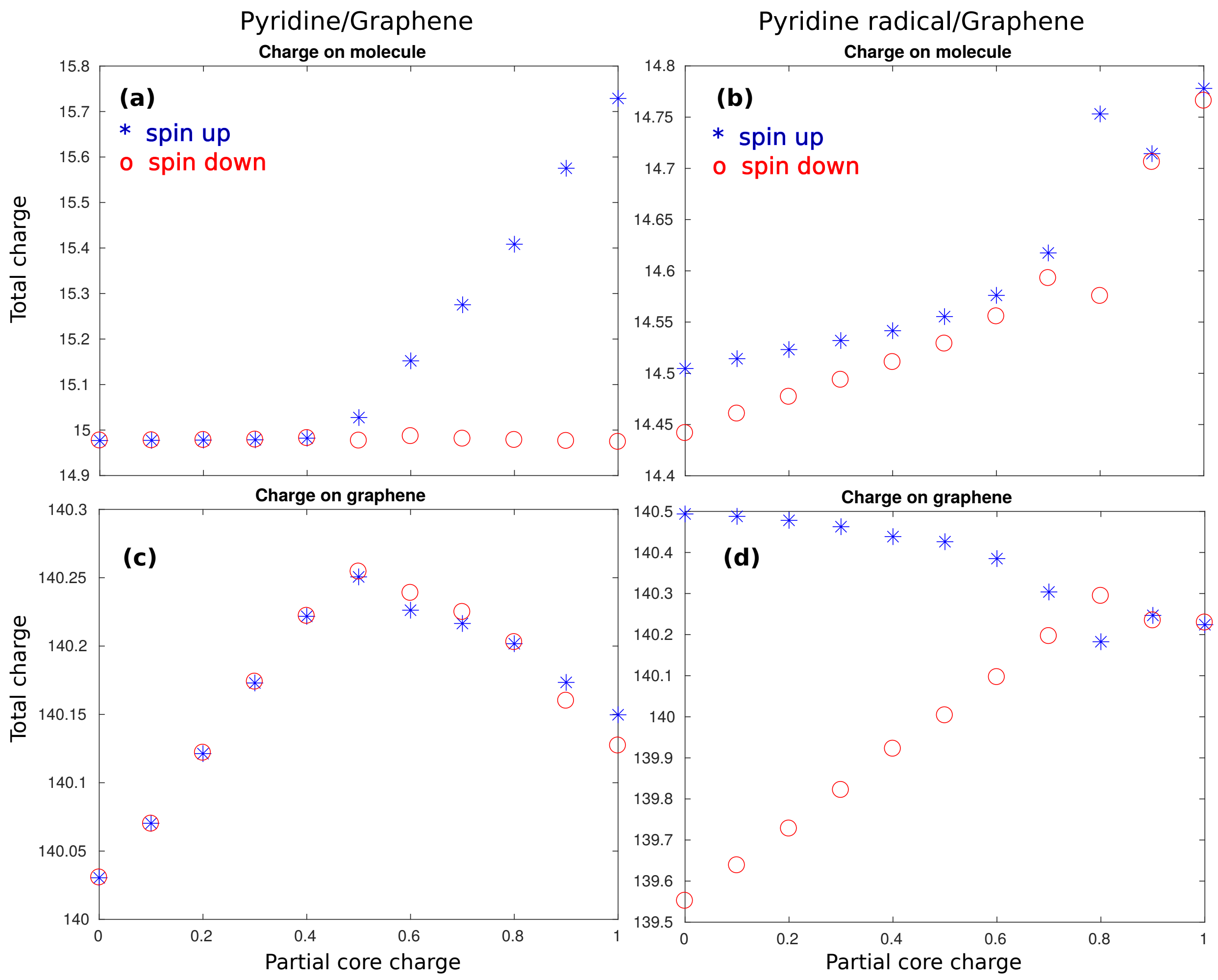

Figure S7. The spin up and spin down charges on the **(a)** molecule and **(c)** graphene for the physisorbed pyridine/graphene case. **(b-d)** are the same for the chemisorbed pyridine radical/graphene case.

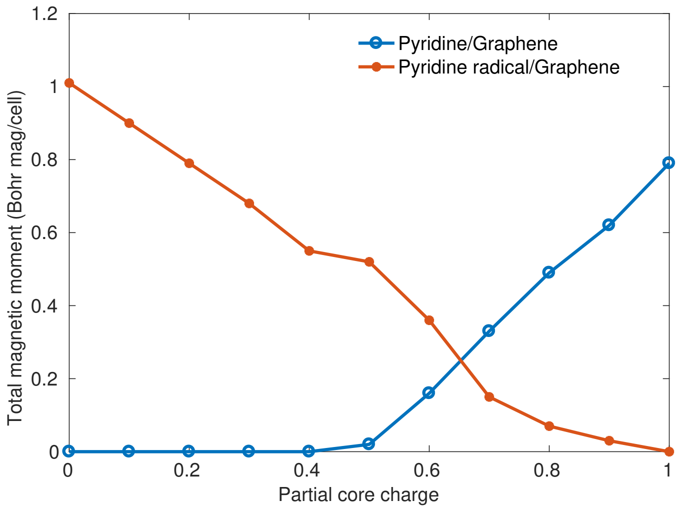

Figure S8. The total magnetic moment of the system for the N 1s excitation for different partial core charges for pyridine/graphene and pyridine radical/graphene cases is shown.
